# Multiple Urethral Hemangiomas Associated with Urethral Stricture: An Uncommon Aetiology for Urethral Bleeding

**DOI:** 10.1155/2019/9071327

**Published:** 2019-02-03

**Authors:** Raquel Varea-Malo, Félix Campos-Juanatey, José Antonio Portillo Martín, Luisana Castillo Carvajal

**Affiliations:** ^1^Urology Department, Marqués de Valdecilla University Hospital, Santander, Spain; ^2^Urology Department, Marqués de Valdecilla University Hospital, Institute of Investigation Marqués de Valdecilla (IDIVAL), Santander, Spain; ^3^Urology Department, Marqués de Valdecilla University Hospital, University of Cantabria, Santander, Spain; ^4^University Foundation of Health Sciences, San Jose Hospital, Bogotá, Colombia

## Abstract

Urethral haemangiomas are rare benign vascular tumours. They usually cause painless urethral bleeding and haematospermia. Urethroscopy is the preferred diagnostic tool, while complementary radiologic studies could describe the extension of the tumour. Treatment should be tailored to each case, considering size, location, and number of lesions. We present a case of a male patient diagnosed with urethral haemangiomas following painless spontaneous urethral bleeding associated with voiding symptoms.

## 1. Introduction

Urethral bleeding aetiology is diverse (traumas, infections, prostatic origin,…), even though vascular malformations are a rare cause. The urethral haemangioma is an uncommon vascular tumour [1] with benign constitution, usually presenting as isolated episodes of urethral bleeding and haematospermia [2]. The preferred diagnostic method is urethrocystoscopy, with identification of haemangiomas typical appearance and with ability to describe size, location, and number of lesions. Due to different clinical presentations, they would require to customize its treatment [3, 4].

Our purpose is to present a case of urethral haemangiomas found in a male. The clinical presentation and diagnostic tests are described. We performed a revision of the existing literature regarding this entity and its treatment.

## 2. Case Report

A 61-year-old man was admitted with acute painless urethral bleeding associated with lower urinary tract symptoms (LUTS), mainly voiding obstruction. He had past medical history of hypertension (HT) and dyslipidemia. When asked about urological backgrounds, the patient described two episodes of acute strong urethral bleeding 20 years ago. Likewise, he underwent surgical primary suture of bleeding areas when haemangiomas of the glans penis were first diagnosed.

**Figure 1:**
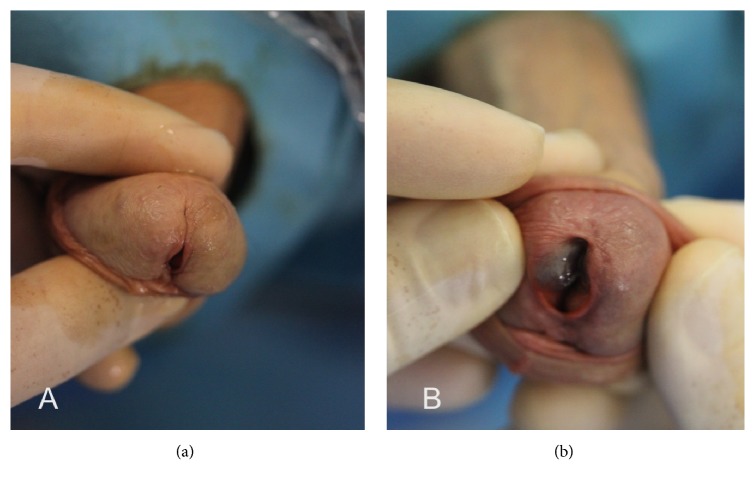
External aspect of glans. (a) External vision, without appreciating hemangioma. (b) Open external urethral meatus, with protrusion of vascular lesion.

**Figure 2:**
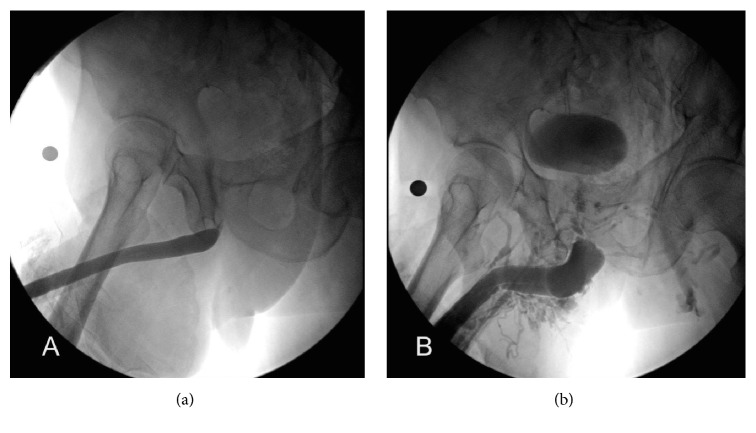
Retrograde urethrogram and voiding cystourethrogram. (a) Proximal bulbar urethral stricture. (b) Contrast extravasation to peribulbar veins.

Physical examination confirmed several haemangiomas in the glandular urethra, located at submeatal level. Those were only visible by gently spreading the external urethral meatus (Figure 1). In addition, we found scrotal haemangiomas and prominent varicose veins in both legs. Digital rectal examination revealed a nonsuspicious medium-sized adenomatous prostate and uroflowmetry showed no value due to a low voiding volume. A short proximal bulbar urethral stricture was diagnosed through a retrograde urethrogram and voiding cystourethrogram (RUG + VCUG). During RUG, contrast extravasation to the peribulbar veins was evident (Figure 2). Urethroscopy confirmed the stricture ring close to the external urinary sphincter.

**Figure 3:**
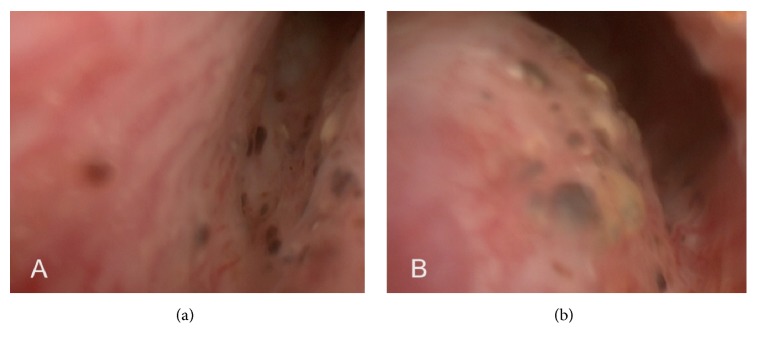
Endoscopic aspect of the prostatic urethra.

After discussion with the patient, an internal urethrotomy under direct vision was the preferred treatment. During this procedure, glans haemangiomas and small haemangiomas in prostatic urethra were identified (Figure 3), unrelated to urethral stricture area. The latter were only visible after opening the strictured segment, but since they did not cause any further bleeding, conservative management was applied. The urethrotomy did not present any haemorrhagic incident, and the catheter was removed on the fifth day after the procedure without any subsequent complications. Follow-up flowmetry evidenced Qmax=12.9 mL/sec, Qmed=5.3 mL/sec with 180 mL of voided volume.

**Figure 4:**
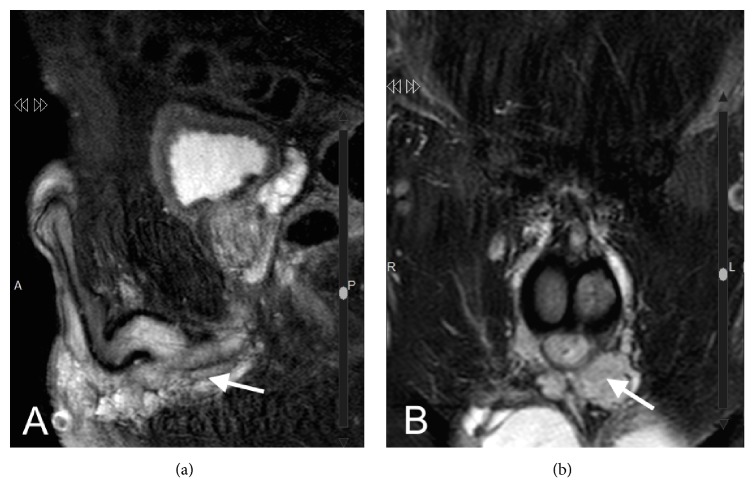
MRI images in T2 sequence, showing periurethral hemangioma. (a) Sagittal section. (b) Coronal section.

Considering endoscopic findings, a pelvic MRI was performed, showing a perineal vascular malformation. This was originated in the urethral bulb and continues through 7 cm of spongy tissue, with up to 14 mm in diameter (Figure 4).

Currently, after almost two years of follow-up, our patient is asymptomatic, with no further episodes of urethral bleeding. In addition, regarding the urethral stricture, our patient is voiding properly presenting adequate uroflowmetry values, with no need for further procedures or dilatations and with a good quality of life.

## 3. Discussion

Urethral haemangiomas are uncommon benign vascular tumours [1]. They were initially described in 1895 [4]. Histologically, they are characterised by vascular spaces covered by endothelium. The aetiology is controversial, being unclear if they are a congenital disease or are caused by vascular neoplasms. Their origin could be related to unusual differentiation of an unipotent angioblastic cell, but other hypotheses were suggested such as herniation of the cavernous bodies' constituent tissue, anomalous revascularization after a traumatism or chronic irritation, and local varicosities [1, 4].

Haemangiomas have been described in other locations, usually skin and liver [5]. Their presentation in the genitourinary system is uncommon, representing around 2% of overall haemangiomas [6]. They can appear in kidney, ureter, bladder, prostate, and urethra, which is the most infrequent location [1, 5].

It is possible to differentiate between different forms of haemangiomas. Those showing a low-flow are venous and cavernous capillary forms, opposed to arteriovenous malformations, which are high flow lesions [6, 7]. The most common haemangioma is the cavernous one [1, 2, 6, 8]. Despite their benign condition, urethral haemangiomas tend to recur despite applied treatments. In contrast, spontaneous regressions have also been reported [9].

Although cases affecting all ages have been described, they are diagnosed more often in males in the third decade of life [4, 5, 7].

Anterior urethral haemangiomas main symptom is urethral bleeding. Nevertheless, when the lesions are located in proximal urethral, they might usually cause haematuria, acute urinary retention, or haemospermia [1, 3, 5, 8]. When these lesions become bigger, they can produce obstructive urinary symptoms and even bulge out throughout external urethral meatus [1]. It is postulated that meatal urethral haemangiomas should bring up their differential diagnosis with condylomata, polyps, malign tumours, abscesses, and caruncles [2].

In our opinion, in spite of the association of both urethral stricture and haemangiomas in the case presented, we consider that there is not any relation between them, since they were not those that caused obstruction to the urinary flow. Due to the self-limited haematuria, we only applied active treatment on urethral stricture, deciding conservative management of urethral haemangiomas.

Urethral haemangiomas could be associated with cutaneous haemangiomas, as in our case, which have scrotal haemangiomas. On the other hand, urethral haemangiomas could be part of Klippel-Trenaunay syndrome, whose three typical features are varicose veins, cutaneous capillary malformations with Port wine colour, and bone and soft tissue hemihypertrophy [10–12]. Urethral haemangiomas affect 3-6% of patients diagnosed with this syndrome [10–12].

The diagnostic procedure of choice is urethrocystoscopy. It should allow identifying and defining the external appearance of lesions, ruling out other urethral bleeding aetiologies. Urethrocystoscopy would as well guide the best therapeutic choice in each case [1, 3]. MRI and doppler ultrasound can also help with diagnosis and characterisation of these lesions in cases with uncertain nature or extension [7].

A high variety of therapeutic options have been proposed for urethral haemangiomas. Treatments based on corticoids, radiotherapy, sclerotherapy, arterial embolization, or photo-ablation with different laser energies (Nd: YAG, argon, KTP, holmium) showed successful results, although urethrectomy could be required in certain cases. Conversely, lesions remaining asymptomatic would not require any treatment [1, 4, 5], as in our case. Location, size, and extension of haemangiomas should be considered, as well as available treatment options in each context. Above all, it is important to consider that urethral haemangiomas may be responsible for urinary obstruction according to their characteristics, although it did not happen in our patient. Therefore, a thorough preoperative planning together with suitable information to the patient is required.

## 4. Conclusion

The urethral haemangioma is an uncommon cause of urethral bleeding, haematospermia, and LUTS. It must be considered during differential diagnosis for these symptoms. The lesions are usually recognisable by urethrocystoscopy, but other imaging studies could be required to get an accurate diagnosis and delimitate their extension. Despite its benign nature, symptomatic cases would require treatment, with a great variety of successful therapeutic options.
